# Development of a machine learning–based sepsis prediction model for real-world clinical settings in South Korea: a single-center retrospective study

**DOI:** 10.7586/jkbns.25.073

**Published:** 2026-02-27

**Authors:** Hye Eun Hwang, Jungmin You, Min Su Kim, Da Young Kim, Jun-Kyu Choi, Hyangkyu Lee

**Affiliations:** 1 College of Nursing and Brain Korea 21 FOUR Project, Yonsei University, Seoul, Korea; 2 Mo Im Kim Nursing Institute, Yonsei University College of Nursing, Seoul, Korea; 3 Small Machines Company, Ltd. Seoul, Korea; 4 Department of Neurology, Ajou University Hospital, Suwon, Korea; 5 Institute for Innovation in Digital Healthcare, Yonsei University, Seoul, Koreea

**Keywords:** Sepsis, Machine learning, Decision support systems, clinical, Electronic health records

## Abstract

**Purpose:**

This study aimed to develop a predictive model for the early identification of patients at risk of sepsis, using routinely available clinical information and laboratory test results collected during the initial phase of patient care.

**Methods:**

This retrospective analysis included electronic medical records of 22,400 adult patients who presented with suspected infection to a tertiary care university hospital in Korea between January 2013 and May 2024. Patients were classified according to Systemic Inflammatory Response Syndrome (score ≥ 2) or Quick Sequential Organ Failure Assessment (score ≥ 2), in combination with sepsis-related International Classification of Diseases, 10th revision codes. Four different machine learning models were trained and validated using five-fold cross-validation. In addition, Shapley additive explanations analysis was performed to interpret the contribution and clinical relevance of key predictive variables.

**Results:**

Among the evaluated models, CatBoost demonstrated the strongest predictive performance. Notably, platelet distribution width, alveolar–arterial oxygen difference, procalcitonin, and the arterial/alveolar oxygen ratio consistently emerged as major predictors. Importantly, several variables that did not reach statistical significance in univariate analysis nevertheless contributed substantially to overall model performance, highlighting the importance of complex, multidimensional interactions among clinical factors.

**Conclusion:**

These findings indicate that a model based on simple, routinely collected clinical data can achieve high predictive accuracy and strong generalizability. Such a tool may support early clinical decision-making by multidisciplinary teams, including nurses, across diverse real-world care settings. Further prospective studies are warranted to validate its clinical utility and to assess its potential effects on patient outcomes.

## INTRODUCTION

### 1. Background

Sepsis is defined as a life-threatening clinical condition marked by multiple organ dysfunction, caused by the host’s impaired response to infection [1]. It represents a notable healthcare burden due to its high morbidity and mortality rates [2]. Early recognition and prompt therapeutic intervention are crucial factors that identify prognosis in sepsis, as delays in treatment increase mortality risk [3]. The mortality rate of patients with sepsis or septic shock is approximately 20%~40% [4], and each hour of delay in antibiotic administration increases the mortality rate by up to 1.8% [5]. Therefore, early and accurate identification of high-risk patients and prompt treatment initiation from the initial presentation are highly recommended.

However, the early assessment of sepsis remains challenging due to its diverse infection routes, broad disease spectrum, frequently non-specific clinical presentations, and often incomplete medical histories [6]. Because no single indicator can define sepsis, various clinical signs, laboratory results, and screening tools are used for early diagnosis and prediction [1,3].

Approaches based on manual surveillance or electronic medical records (EMRs) may lack sensitivity and specificity, and their performance can vary depending on the clinical setting [7,8]. Blood cultures are the standard diagnostic test for detecting bloodstream infections; however, their low sensitivity and long turnaround time limit their usefulness for early diagnosis [9].

These limitations pose challenges to distinguish sepsis from non-sepsis, non-infectious systemic immune responses. Systemic immune activation in sepsis is triggered by infectious agents (pathogens), whereas non-infectious systemic immune responses can occur due to trauma, postoperative states, acute pancreatitis, or other non-infectious triggers [1]. Differentiating patients with sepsis based solely on clinical signs is difficult in the absence of identified pathogens.

Recently, artificial intelligence and machine learning (ML) methods have been increasingly applied to improve disease prediction accuracy by learning complex patterns from large-scale clinical data. In particular, ML-based prediction models that integrate diverse laboratory results and clinical data can overcome the limitations of conventional screening tools [10]. Consequently, early prediction models have been reported in various healthcare settings internationally [11,12]. In Korea, a few studies have aimed to improve sepsis prediction performance using initial emergency department data [13,14]. However, most domestic studies remain focused on patients admitted to the intensive care unit (ICU), and research encompassing the early management phase across diverse clinical settings remains insufficient.

Moreover, clinical decision-making using complex prediction models can be limited when only a small amount of clinical information is available at the initial presentation.

For example, prior ICU-based prediction models have commonly included continuously monitored vital signs, such as real-time blood pressure, heart rate, respiratory rate, and oxygen saturation, because high-frequency physiologic data can enhance early detection of clinical deterioration [15]. However, such continuous monitoring is rarely available in non-ICU settings where initial assessments typically occur. Accordingly, models that maintain strong predictive performance using only routinely collected laboratory and clinical data are required.

Therefore, we aimed to develop and validate an ML model for early sepsis prediction using routinely collected EMR and laboratory data available at the initial point of care. Additionally, by examining the clinical significance of key predictive variables, this study sought to enhance interpretability and support future integration into clinical decision-support systems.

### 2. Study aim

This study aims to identify clinical factors associated with sepsis and to develop and validate an ML-based algorithm for early diagnosis. The specific objectives are as follows:

1) To develop a predictive model for sepsis using ML algorithms.

2) To investigate the characteristics of clinical variables included in the developed prediction model and compare their relative importance.

## METHODS

### 1. Study design

This retrospective secondary data analysis aims to identify risk factors for sepsis and develop an ML-based predictive model by selecting sepsis and non-sepsis groups among patients suspected of infection who visited a general hospital.

### 2. Participants

This study screened 31,148 adult encounters evaluated for possible acute infection at Severance Hospital, Yonsei University, from January 1, 2013, to May 3, 2024. Because sepsis and non-sepsis presentations often arise in different clinical contexts, the two groups in this study were not derived from a single unified cohort, but were identified through distinct clinical pathways.

In routine clinical practice, physiological abnormalities meeting the Systemic Inflammatory Response Syndrome (SIRS) criteria (score ≥ 2) or the quick Sequential Organ Failure Assessment (qSOFA) criteria (score ≥ 2) frequently trigger evaluation for potential sepsis. In this study, these indicators were applied within each selection pathway rather than to define a single pooled cohort.

**Figure 1.**
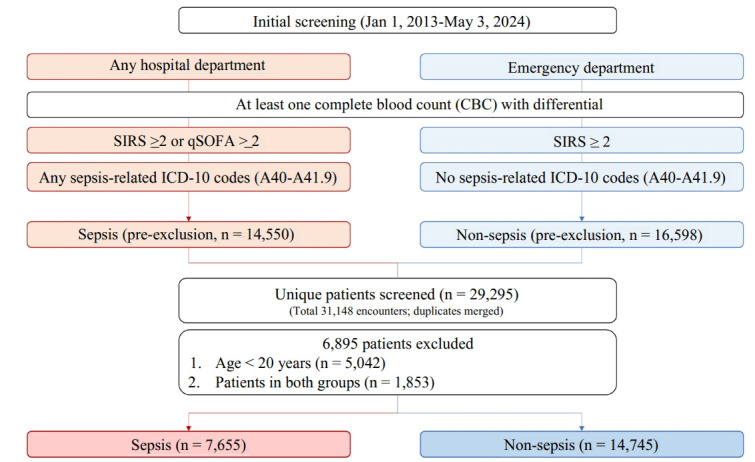
Flow chart of the study population. SIRS = Systemic Inflammatory Response Syndrome; qSOFA = quick Sequential Organ Failure Assessment; ICD-10 = International Classification of Diseases, 10th Revision.

After excluding individuals aged < 20 years and patients appearing in both groups because of multiple encounters, 22,400 patients remained eligible (Figure 1).

#### 1) Sepsis group

The sepsis group included adult patients from any hospital department who had at least one complete blood count (CBC) with differential during the initial phase of care. Patients with SIRS ≥ 2 or qSOFA ≥ 2 were considered clinically suspected of sepsis, and those assigned any of the 24 sepsis-related International Classification of Diseases, 10th Revision (ICD-10) codes (A40–A41.9) were operationally classified as sepsis cases.

Although Sepsis-3 emphasizes organ dysfunction and SOFA scoring [1], early recognition in emergency and outpatient environments often relies on SIRS because of its higher sensitivity [1,3,7,16]. Therefore, both SIRS and qSOFA served as pathways of clinical suspicion, with ICD-10 coding functioning as the definitive classification criterion.

#### 2) Non-sepsis group

The non-sepsis group comprised patients who visited the emergency department with fever or hypothermia as their chief complaint, had results available for CBC with differential and met the SIRS of ≥ 2 criterion, but did not meet the diagnostic criteria for sepsis, and were not assigned any of the sepsis-related ICD-10 codes.

The non-sepsis group was based on emergency department patients, while the sepsis group was defined patients across the hospital. Although this approach may not be ideal for a typical research design, it was chosen to reflect the fact that patients suspected of sepsis but not diagnosed with it are often found in the emergency department [16]. This consideration was incorporated into the study design, as it was believed that this would capture clinically meaningful results for model training.

Only SIRS was applied as an explicit criterion in this group to ensure inclusion of acutely ill patients exhibiting systemic inflammatory responses, which aligns with how many non-septic acute conditions initially manifest. qSOFA was not used as an exclusion criterion; therefore, some patients with elevated qSOFA scores may have been included in the control cohort.

This design was informed by expert consensus, which emphasized that using SIRS ≥ 2 to define the control population enhances the model’s clinical applicability because SIRS remains a common initial screening trigger in many real-world settings. Accordingly, the control group intentionally included both patients who might initially prompt sepsis screening (e.g., elevated qSOFA or abnormal physiology) and those with less severe acute presentations, provided they did not ultimately meet sepsis criteria or receive sepsis-related ICD-10 codes.

By allowing this heterogeneity, the control group more accurately reflected the real-world spectrum of patients evaluated for suspected sepsis and enabled the predictive model to distinguish true sepsis from other acute states with overlapping early physiological abnormalities.

This study conducted a secondary data analysis based on EMRs, and biological sex was used as a variable.

### 3. Instruments

Major clinical variables to be included in the model were identified through consultation with a multidisciplinary panel of 10 experts, including physicians from internal medicine, laboratory medicine, and family medicine; nursing faculty; and specialists in predictive modeling. The panel reviewed evidence on sepsis-related predictors and determined clinically relevant and routinely available variables for model inclusion [1,3].

Variables were excluded when considered redundant, clinically insignificant, or insufficiently available in the EMRs. Chronic respiratory, renal, hepatic, and cardiovascular diseases, malignancy, and immunosuppression were initially evaluated as separate predictors [1–4,17–21]; however, multimorbidity was ultimately represented using the Charlson Comorbidity Index (CCI) and age-adjusted CCI to reduce dimensionality and multicollinearity while retaining prognostic information [17–19].

Vital signs were excluded due to substantial EMR quality issues. Measurement frequency varied across settings, many values were missing or irregular, and timestamps often misaligned with laboratory tests, making reliable temporal comparison impossible. To avoid introducing measurement bias—consistent with recommendations against including poorly recorded or overlapping predictors [22–25]—vital signs and neurologic variables used in SIRS and qSOFA screening were not incorporated as predictors. Instead, organ dysfunction was captured through more reliably recorded laboratory and arterial blood gas analysis (ABGA) variables.

The final list of included variables is provided in Appendix Table 1.

#### 1) General characteristics

Data including age, sex, primary diagnosis, and comorbidities were collected based on the initial visit. The number of comorbid conditions was reflected in the CCI score.

The CCI was calculated based on the original scoring system proposed by Charlson et al. [17], and comorbid conditions were defined using the ICD-10 coding algorithm developed by Quan et al. [18]. Regarding age adjustment, as proposed by Charlson et al. [17], 1 point was added for each decade over 40 years and the age-adjusted CCI was obtained by summing the original CCI score and the age-related score.

The age-adjusted CCI was used to better capture the combined effects of age and comorbidities on patient prognosis. Because age acts as a risk factor for chronic diseases, incorporating it into the CCI allows for a more accurate representation of patient health status and comorbidity burden. Notably, the age-adjusted CCI has also demonstrated prognostic relevance in recent sepsis cohorts, supporting its clinical utility as a comorbidity indicator [19].

#### 2) Diagnostic characteristics

Diagnostic characteristics included blood test results such as CBC clinical chemistry including serum electrolytes, and ABGA These are commonly used to evaluate infection severity and the patient’s physiological status, as referenced in studies on sepsis prediction [1,3]. Continuous data were extracted for at least 3 days before sepsis diagnosis.

### 4. Data collection

Data were collected through a standardized extraction procedure from the institutional EMR system. Eligible patients diagnosed during the study period (January 1, 2013–May 3, 2024) were identified according to predefined criteria. For each case, clinical records within the first 3 days before diagnosis were systematically retrieved, including demographics, comorbidities, laboratory results (Appendix Table 1). All patient data were anonymized before analysis. This retrospective data collection process was conducted after approval by the Institutional Review Board (IRB) and the Data Review Board (DRB).

### 5. Statistical analysis

The binary variables were presented as counts and percentages and were assessed using the chi-square test or Fisher’s exact test. Normality of continuous variables was assessed using the Shapiro–Wilk test. As continuous variables did not conform to a normal distribution, all continuous variables were compared between groups using the Mann–Whitney U test. Continuous variables are presented as mean ± standard deviation. *P*-values of < .05 indicated statistical significance. The statistical analyses were conducted using Python 3.13 (Python Software Foundation, Wilmington, DE, USA) and respective libraries.

#### 1) Data preprocessing

A preprocessing pipeline was applied to the final cohort of 22,400 patients. Major challenges included long-tailed sequence lengths and substantial missingness in clinical data [26]. Sepsis status served as the target variable, and all other clinical variables were treated as independent numeric features [27]. Missing values in continuous variables were imputed with medians [22], and features were standardized using StandardScaler [28]. These steps reduced bias and variance across features and improved model stability during training [29].

#### 2) Predictive modeling

Four ML approaches were developed and compared: CatBoost, XGBoost, a Transformer-based deep learning model, and a Soft Voting Ensemble model. Each algorithm was selected for its capacity to capture complex non-linear associations while incorporating intrinsic mechanisms to mitigate overfitting. Specifically, CatBoost employs ordered boosting and built-in regularization, which have reduced variance and improved generalizability in clinical prediction tasks [30]. XGBoost integrates shrinkage, subsampling, and L1/L2 regularization, providing strong protection against overfitting and exhibiting excellent predictive accuracy in early sepsis detection [31]. The Transformer-based deep learning model was optimized with dropout layers, weight decay, and early stopping criteria to prevent overfitting while effectively modeling long-term dependencies in time-series data [32]. Finally, the Soft Voting Ensemble model aggregated probabilistic predictions from the individual classifiers to improve stability and reduce model-specific variance, an approach shown to improve diagnostic robustness in other domains, including oncology [33].

#### 3) Model validation and performance evaluation

No resampling or weighting adjustments were applied to address class imbalance. The dataset was partitioned into a training set (70%) and a test set (30%). Five-fold stratified group cross-validation was performed, keeping all observations from each patient within a single fold to prevent information leakage and preserve class balance [23,24]. Nested cross-validation scheme with grid search was applied to rigorously tune hyperparameters and prevent optimistic bias [25]. Together with regularization, dropout, and ensemble methods, these procedures improved generalizability and reduced overfitting [34,35].

Model performance was assessed using accuracy, sensitivity (recall), specificity, precision, and F1-score [36,37]. A crucial metric was the area under the receiver operating characteristic curve (AUC), which summarizes the trade-off between the true positive rate and the false positive rate to provide a single measure of the model’s discriminative power [38]. To ensure interpretability, SHAP (SHapley Additive exPlanations) was employed, utilizing summary dot plots to visualize both global feature importance and the distribution of SHAP values across samples, thereby highlighting the influence of individual predictors [39]. Python 3.13 (Python Software Foundation, Wilmington, DE, USA) and associated scientific libraries were used for all analyses.

### 6. Ethical considerations

This research was conducted following review and approval by the IRB and the affiliated DRB of Severance Hospital, Yonsei University Health System (Approval No.: 4-2023-1521). As a retrospective study, it used EMRs obtained during routine clinical care, and all personally identifiable information was anonymized during the analysis process.

The collected EMR data were stored in a designated independent cloud environment within the institution’s Digital Health Center, and all analyses were strictly performed within this secure platform. Data remained on the on-site system and were accessible only to the designated analyst in the research team. Data management was overseen by the principal investigator, with regular password updates done to prevent unauthorized access or leakage. Only the final algorithm generated through data analysis was permitted to be exported outside the institution.

The data will be utilized solely for research purposes during the approved study period. After study completion, the data will be retained for 3 years to ensure reliability and then permanently destroyed.

## RESULTS

### 1. Demographic and clinical characteristics

This study screened 31,148 adult encounters related to acute infection. Of these, 22,400 patients were included and classified into two groups—the sepsis group and the non-sepsis group—each defined through distinct clinical pathways. Table 1 summarizes the baseline demographic and clinical characteristics of the patients.

The mean age of the sepsis group was 65.73 ± 15.19 years, which was higher than that of the non-sepsis group (60.09 ± 17.14 years). Furthermore, the proportion of male patients was higher in the sepsis group (56.6%) than in the non-sepsis group (51.1%). The occurrence of sepsis indicated statistically significant differences in terms of age, sex, CCI score, and age-adjusted CCI score.

The CBC revealed marked differences in hemoglobin, hematocrit, red blood cell count, mean corpuscular hemoglobin concentration, mean corpuscular hemoglobin, red cell distribution width, white blood cell (WBC) count, neutrophil percentage, lymphocyte percentage, monocyte percentage, platelet count, mean platelet volume (MPV), platelet distribution width (PDW), neutrophil-to-lymphocyte ratio (NLR), and platelet-to-lymphocyte ratio.

In clinical chemistry tests, total bilirubin, aspartate transaminase (AST), creatinine, lactate, and C-reactive protein (CRP) levels markedly differed between the sepsis and non-sepsis groups.

ABGA revealed marked differences in pH, bicarbonate (HCO_3_^-^), base excess in extracellular fluid, alveolar–arterial oxygen difference (AaDO_2_), arterial-to-alveolar oxygen ratio (a/A ratio), PaO_2_/FiO_2_ ratio, arterial oxygen content (CaO_2_), and total carbon dioxide (TCO_2_). Furthermore, peripheral oxygen saturation (SpO_2_) and the time from diagnosis to admission markedly differed between the two groups.

### 2. Performance of prediction models

**Figure 2.**
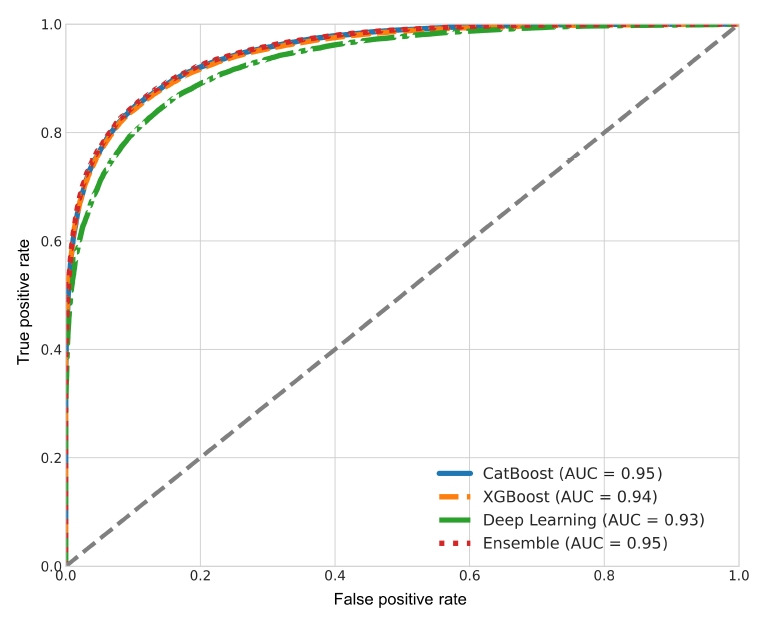
AUC-receiver operating characteristics curves of assessed models. AUC = Area under the curve.

Table 2 and Figure 2 present the predictive performance of the four developed models. The CatBoost model demonstrated high sensitivity, achieving a sensitivity of .92, specificity of .79, precision of .78, F1-score of .84, and an AUC of .95. The XGBoost model generated a sensitivity of .91, specificity of .81, precision of .79, F1-score of .85, and an AUC of .94. The deep learning model demonstrated the highest specificity and precision, with a sensitivity of .79, specificity of .91, precision of .87, F1-score of .83, and an AUC of .93. Figure 2 illustrates the ROC curves for all four models, indicating that their AUC values cluster in a high range (.93~.95) and that the curves for CatBoost, XGBoost, and the ensemble almost overlap, reflecting very similar overall discriminative performance.

The proposed soft voting ensemble model attained a sensitivity of .91, specificity of .83, precision of .80, an F1-score of .85, and an AUC of .95, providing a slightly improved balance between sensitivity and specificity compared with the single models. However, the gains in F1-score and AUC over CatBoost and XGBoost were minimal (≤ .01~.02). In line with the Occam’s razor–inspired parsimony principle in ML, which recommends preferring simpler models when predictive performance is comparable [40,41], and given the strong performance and intrinsic regularization of CatBoost and XGBoost described above [30,31], we selected these two models as the primary models for subsequent analyses and SHAP-based variable importance assessment.

### 3. Variable importance

**Figure 3.**
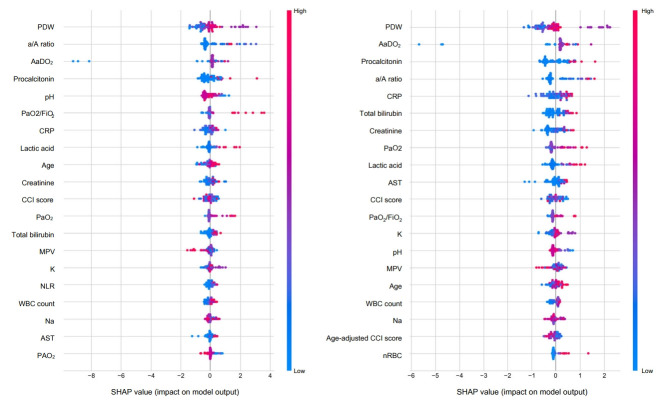
SHAP analysis results (left: XGBoost; right: CatBoost). SHAP = SHapley Additive exPlanations; PDW = Platelet distribution width; a/A ratio = Arterial-to-alveolar oxygen ratio; AaDO_2_ = Alveolar–arterial oxygen difference; CRP = C-reactive protein; CCI = the Charlson Comorbidity Index; MPV = Mean platelet volume; NLR = Neutrophil-to-lymphocyte ratio; AST = Aspartate transaminase; WBC = White blood cell; nRBC = Nucleated red blood cell.

To interpret these models and identify the most influential clinical variables, SHAP analysis was conducted. SHAP summary plots highlight the top 20 most influential variables for the XGBoost (Figure 3, left) and CatBoost (Figure 3, right) models. With features ordered by global importance, high feature values (indicated in red) distributed to the right signify a positive contribution to the predicted probability of sepsis, whereas those distributed to the left indicate a negative contribution [39].

Across both models, the most influential variables included PDW, a/A ratio, AaDO_2_, procalcitonin, pH, CRP, PaO_2_/FiO_2_, total bilirubin, creatinine, lactic acid, age, AST, CCI score, K, MPV, WBC count, and Na. In general, higher values of PDW, procalcitonin, CRP, PaO_2_, total bilirubin, creatinine, lactic acid, age, AST, K, and WBC count were associated with an increased predicted probability of sepsis, whereas higher a/A ratio, pH, and MPV were associated with a decreased predicted probability. CCI score and Na showed mixed patterns of contribution across models, and NLR and PAO_2_ were identified as top variables only in one of the two models. Taken together, these findings highlight the central role of hematologic, inflammatory, and respiratory parameters in distinguishing sepsis from other acute conditions.

## DISCUSSION

This study identified factors associated with sepsis and developed a predictive model using ML algorithms. It primarily aimed to facilitate clinical decision-making and provide foundational evidence for efficient resource allocation by enabling the early identification of sepsis.

In this study, three ML algorithms were evaluated as primary candidates. Among the individual models, CatBoost and XGBoost exhibited the most robust performance, particularly in terms of AUC and sensitivity, surpassing the deep learning model. While the ensemble approach yielded similar results, these two models were prioritized for their parsimony and interpretability. A previous study reported AUCs of 0.87 in non-ICU patients using a CBC-only model [42], 0.87 in general inpatient settings [43], and 0.93 in an emergency department setting [44]. In contrast, our model exhibited superior performance with an AUC of 0.95.

Although direct comparison across studies is inherently limited because the types of input variables, clinical settings, and measurement frequency differ substantially, several major predictors identified in this study were generally consistent with those reported in previous sepsis research. For example, platelet distribution–related indices (PDW, MPV), oxygenation markers, and organ dysfunction indicators such as bilirubin and creatinine have been repeatedly highlighted for their diagnostic or prognostic value in studies conducted in ICU, emergency department, and general-ward settings, including those using ML-based approaches [1,3,13,14,15,42,45].

Similarly, a recent meta-analysis [15] reported that ICU-based sepsis prediction models frequently relied on predictors reflecting these same physiological domains—such as platelet count, lactate, WBC count, hemoglobin, and PaO_2_—alongside ICU-specific indicators such as SOFA scores and ICU length of stay. Although our model used only routinely collected laboratory tests and excluded ICU-specific variables, its top predictors converged on these well-established physiological domains, supporting the clinical plausibility of the model.

SHAP analysis clarified how these variables contributed to model predictions. In general, most predictors with positive SHAP contributions also had higher values in the sepsis group in univariate analyses. However, several key variables did not show statistically significant between-group differences or were even higher in the non-sepsis group, suggesting that SHAP captured patterns that were not apparent from traditional mean comparisons. For example, procalcitonin was identified as an important predictor in both CatBoost and XGBoost, consistent with previous literature [46], despite its lack of statistical significance in the univariate analysis.

Hematologic markers illustrated this pattern particularly well. They ranked among the top predictors and generally aligned with the expected immune response in sepsis—characterized by neutrophilia and lymphopenia [20,21]. Even so, several leukocyte subtypes and electrolyte markers (Na and K) showed mixed or unexpected directions when comparing SHAP contributions with group-level means. These variations likely reflect clinical factors such as differences in disease severity, sampling times, early antibiotic exposure, or other treatment-related influences [1,45].

A similar divergence emerged in oxygenation-related variables. Oxygen-transport indicators were highly influential in SHAP analyses, yet their contributions contrasted with the conventional expectation that oxygenation typically declines in sepsis [1]. In our cohort, hemoglobin and hematocrit were higher in the non-sepsis group, whereas PaO_2_ and PaO_2_/FiO_2_ were higher in the sepsis group—a pattern likely shaped by oxygen therapy or ventilatory support rather than intrinsic physiological differences. This highlights how treatment context can shift group-level values while individual-level contributions remain predictive.

Inflammatory markers showed the same kind of discrepancy. CRP, in particular, increased the predicted probability of sepsis in SHAP analysis, even though mean CRP levels were higher in the non-sepsis group. This paradox may be explained by cohort heterogeneity and immunologic dynamics: non-sepsis patients may have had localized infections or acute inflammatory responses, whereas some sepsis patients may have been captured during an immunosuppressive phase associated with organ dysfunction [1]. Moreover, sepsis often follows a biphasic course—an early hyperinflammatory phase followed by immunosuppression—during which CRP may decline despite ongoing infection [20,47].

Taken together, these findings suggest that even when mean values deviate from expected patterns, SHAP analysis reveals individual-level signals that remain strongly predictive of sepsis.

Overall, the major variables identified in this study were broadly consistent with known clinical characteristics of sepsis. However, several important predictors did not show statistical significance or exhibited higher values in the non-sepsis group, reflecting patient heterogeneity and treatment-related effects [20,21]. These discrepancies reinforce that ML models can capture clinically meaningful interactions beyond simple group-level comparisons and highlight their utility as decision-support tools [14].

However, certain tests are selectively ordered in sicker patients, leading to prediction biases due to disease severity and resource availability. Although the model reflects real-world clinical practice, false negatives in sepsis prediction carry substantial risk [45]; thus, it should support rather than replace clinical judgment, and standard sepsis management must remain prioritized in high-risk cases.

A key contribution of this work is the development of a generalizable model that can be implemented across the hospital, regardless of the unit in which the patient is first assessed. Unlike many previous studies that focused on ICU populations [15], employed large feature sets [12,48], or restricted their scope to short prediction windows [49,50], our study included patients from diverse non-ICU units and used a parsimonious set of routinely collected laboratory tests and basic clinical data. Because continuous vital-sign monitoring is rarely available in general wards or emergency departments, achieving strong predictive performance without high-frequency physiological data represents a practical advantage of our model. Thus, the strength of our approach lies not in methodological superiority over ICU-based models but in its applicability under non-ICU constraints and its potential for implementation across diverse clinical environments.

From a nursing perspective, the model may enhance patient safety by supporting early recognition of deterioration in settings with limited monitoring capacity. Nurses can play a crucial role in identifying early signs of sepsis, drawing not only on laboratory results but also on subtle clinical cues. Because the model performs effectively using only routinely collected laboratory data, it could serve as a complementary decision-support tool in resource-constrained environments. However, vital signs remain foundational in nursing assessment, and the absence of them represents an important limitation. Future work should incorporate more complete vital-sign information as documentation improves, ensuring closer alignment with the comprehensive assessments conducted by nurses.

This research has several limitations. First, as the data were collected from a single institution, the generalizability of the findings may be limited. Moreover, the definition of sepsis in this study was based on SIRS or qSOFA with sepsis-related ICD-10 codes rather than the Sepsis-3 consensus criteria, which emphasize organ dysfunction and SOFA scoring. As a result, some patients who did not strictly meet the Sepsis-3 definition may have been included in the sepsis group. While this represents a methodological limitation, it also reflects the clinical reality in many emergency and outpatient settings, where early organ dysfunction assessment is not always feasible and SIRS- or qSOFA-based criteria remain widely used for initial triage and clinical decision-making.

Second, the construction of the sepsis and non-sepsis groups was not fully symmetrical. The sepsis group included patients from all hospital settings, whereas the non-sepsis group was restricted to emergency department patients. This asymmetry may introduce selection bias and affect model calibration, as the two groups may differ not only in disease status but also in care environment, illness severity, and documentation practices. Although this design reflects real-world triage—where many patients initially suspected of sepsis but ultimately not diagnosed with it are encountered in the emergency department—it warrants cautious interpretation. This asymmetric cohort construction remains a clear methodological limitation and may have introduced systematic selection bias that influenced group comparability and model performance.

Third, sepsis diagnoses were based on ICD-10 codes assigned by physicians, which may include classification errors or inaccuracies due to incomplete clinical data, as a retrospective secondary data analysis. Furthermore, many variables were excluded due to missing data. However, this study primarily intended to develop a practical and generalizable model using commonly available clinical tests rather than to target a specific patient population, and thus it meets its original objective. Still, variable availability, missing data handling, and class imbalance may have affected the performance, requiring external validation and calibration. Fourth, this study assessed only classification performance and did not examine clinical outcomes, including survival rates or length of hospital stay.

## CONCLUSION

In this study, ML-based models were developed and validated using routinely collected clinical data to enable early identification of sepsis in real-world hospital environments. CatBoost and XGBoost achieved strong and comparable discriminative performance, demonstrating that robust prediction is feasible without ICU-specific or continuously monitored variables. By capturing multidimensional clinical patterns, the proposed model can be implemented across emergency departments, outpatient clinics, and general wards without requiring additional customization. These characteristics highlight its potential utility as a practical and scalable decision-support tool for early risk stratification and timely clinical intervention. Furthermore, the reliance on routinely available laboratory data enhances compatibility with existing workflows and may support safer and more consistent triage in settings with limited monitoring capacity. External validation and prospective studies are warranted to confirm generalizability, assess clinical impact, and guide integration into standard sepsis management pathways.

## Figures and Tables

**Table 1.** Demographic and Clinical Characteristics (N = 22,400)

Variables	Sepsis (n = 7,655)	Non-sepsis (n = 14,745)	χ² or Z	*p*
Demographic characteristics				
Age (years)	65.73 ± 15.19	60.09 ± 17.14	-23.20	< .001
Sex				
Men	4,329 (56.6)	7,530 (51.1)	60.59	< .001
Women	3,326 (43.4)	7,215 (48.9)		
CCI score	3.02 ± 3.13	2.69 ± 3.07	-9.81	< .001
Age-adjusted CCI score	5.23 ± 3.30	4.48 ± 3.42	-17.72	< .001
Initial laboratory findings				
CBC				
Hb (g/dL)	10.78 ± 2.30	11.58 ± 2.27	19.30	< .001
Hct (%)	32.05 ± 6.69	34.31 ± 6.59	18.75	< .001
RBC (×10⁶/μL)	3.52 ± 0.77	3.77 ± 0.77	18.18	< .001
MCHC (g/dL)	33.62 ± 1.34	33.75 ± 1.26	5.14	< .001
MCH (pg)	30.79 ± 2.47	30.88 ± 2.47	2.84	.005
MCV (fL)	91.58 ± 6.76	91.52 ± 6.64	-0.07	.947
RDW (%)	15.19 ± 2.58	14.66 ± 2.25	-11.41	< .001
WBC (×10³/μL)	10.48 ± 7.77	10.40 ± 9.51	-0.97	.331
Neutrophils (%)	76.67 ± 17.46	77.95 ± 17.87	5.89	< .001
Lymphocytes (%)	14.32 ± 15.05	13.56 ± 14.49	-3.48	< .001
Monocytes (%)	6.04 ± 4.54	5.39 ± 4.52	-11.08	< .001
Eosinophils (%)	1.64 ± 2.85	1.22 ± 1.93	-3.15	.002
Basophils (%)	0.31 ± 0.33	0.32 ± 0.40	0.43	.669
nRBC (%)	0.35 ± 2.45	0.09 ± 1.37	-31.73	< .001
Platelet count (×10⁹/L)	212.77 ± 131.27	209.9 ± 123.49	-0.62	.536
MPV (fL)	9.28 ± 1.47	8.52 ± 1.35	-28.52	< .001
PDW (%)	55.47 ± 9.81	53.77 ± 9.65	-8.03	< .001
NLR	57.13 ± 1864.82	49.90 ± 1814.87	3.82	< .001
PLR	102.92 ± 3501.87	141.51 ± 6807.86	4.49	< .001
Clinical chemistry				
Total bilirubin (mg/dL)	2.26 ± 4.26	1.18 ± 1.74	-4.81	< .001
AST (IU/L)	58.32 ± 85.99	82.34 ± 371.91	-2.89	.004
Creatinine (mg/dL)	1.34 ± 1.45	1.14 ± 1.27	-0.96	.337
Sodium (mmol/L)	135.56 ± 5.93	135.61 ± 4.92	-0.06	.951
Potassium (mmol/L)	4.07 ± 0.67	4.13 ± 0.57	2.18	.029
Lactic acid (mmol/L)	2.70 ± 2.69	1.80 ± 1.53	-14.17	< .001
Procalcitonin (ng/mL)	6.69 ± 19.08	5.25 ± 14.22	-0.53	.599
CRP (mg/L)	86.49 ± 78.95	113.90 ± 91.44	4.48	< .001
ABGA				
pH	7.42 ± 0.10	7.45 ± 0.16	18.78	< .001
PaO_2_ (mmHg)	96.17 ± 46.57	85.92 ± 29.96	-8.02	< .001
PaCO_2_ (mmHg)	32.93 ± 11.17	29.18 ± 6.33	-17.94	< .001
HCO_3_^-^ (mmol/L)	21.22 ± 5.82	20.51 ± 3.97	-8.30	< .001
BE-B (mmol/L)	-2.29 ± 5.78	-2.00 ± 3.65	-0.69	.490
BE-ECF (mmol/L)	-3.45 ± 6.51	-3.62 ± 4.25	-4.53	< .001
tHb (g/dL)	11.17 ± 2.45	11.36 ± 2.21	4.81	< .001
PAO_2_ (mmHg)	111.76 ± 24.03	114.01 ± 9.22	15.26	< .001
AaDO_2_ (mmHg)	36.21 ± 22.24	32.00 ± 20.31	-6.40	< .001
a/A ratio	30.49 ± 58.50	10.41 ± 27.00	-27.11	< .001
PaO_2_/FiO_2_	453.78 ± 217.75	410.53 ± 142.66	-6.78	< .001
CaO_2_ (mL/dL)	14.86 ± 3.40	15.27 ± 3.02	6.61	< .001
SBC (mmol/L)	22.67 ± 4.71	22.75 ± 3.14	-0.79	.431
TCO_2_ (mmol/L)	22.24 ± 6.05	21.40 ± 4.13	-8.94	< .001
Clinical information				
SpO_2_ (%)	94.49 ± 8.48	95.13 ± 6.76	-0.94	.345
Time from diagnosis to admission (days)	-2.00 ± 2.11	-0.63 ± 0.77	34.94	< .001

Values are presented as the mean ± standard deviation or n (%). CCI score = Charlson comorbidity index; CBC = Complete blood count; Hb = Hemoglobin; Hct = Hematocrit; RBC = Red blood cell count; MCHC = Mean corpuscular hemoglobin concentration; MCH = Mean corpuscular hemoglobin; MCV = Mean corpuscular volume; RDW = Red cell distribution width; WBC = White blood cell count; nRBC = Nucleated red blood cell; MPV = Mean platelet volume; PDW = Platelet distribution width; NLR = Neutrophil-to-lymphocyte ratio; PLR = Platelet-to-lymphocyte ratio; AST = Aspartate aminotransferase; CRP = C-reactive protein; ABGA = Arterial blood gas analysis; pH = Hydrogen ion concentration; PaO_2_ = Arterial oxygen partial pressure; PaCO_2_ = Arterial carbon dioxide partial pressure; HCO₃⁻ = Bicarbonate; BE-B = Base excess in blood; BE-ECF = Base excess in extracellular fluid; tHb = Total hemoglobin; PAO_2_ = Calculated alveolar oxygen partial pressure; AaDO_2_ = Alveolar–arterial oxygen difference; a/A ratio = Arterial/alveolar oxygen ratio; PaO_2_/FiO_2_ = Partial pressure of arterial oxygen to the fractional inspired oxygen; CaO_2_ = Arterial oxygen content; SBC = Standard bicarbonate concentration; TCO_2_ = Total carbon dioxide; SpO_2_ = Peripheral oxygen saturation.

**Table 2.** Model Performance

Model	Accuracy	Sensitivity	Specificity	Precision	F1-score	AUC
CatBoost	.85	.92	.79	.78	.84	.95
XGBoost	.85	.91	.81	.79	.85	.94
Deep learning	.86	.79	.91	.87	.83	.93
Ensemble	.86	.91	.83	.80	.85	.95

F1-score = Harmonic mean of precision and recall; AUC = Area under the curve.

## Data Availability

Please contact the corresponding author for data availability

## Appendix Table 1.

**Table:** Mapping of Guideline-derived Candidate Predictors to Final Predictors in the Sepsis Prediction Model

Predictor domain	Sepsis-related predictors	Selection status	Final predictors in the sepsis prediction model	Justification for modification or exclusion^†^
Demographics	Age, sex [1,2,4]	Retained	Age, sex	
Comorbidities	Chronic respiratory disease, chronic kidney disease, chronic liver disease, chronic cardiovascular disease [1–4,17–19]	Modified	CCI score	Summarized by the CCI and age-adjusted CCI to reduce dimensionality and multicollinearity while capturing the combined impact [17-19] of multimorbidity and age on sepsis risk [18]
	Malignancy / immunosuppression [1,3,17,44,45]		Age-Adjusted CCI score	
Vital signs	SBP, MAP, heart rate, respiratory rate, body temperature [1,3,7,8,12,13]	Excluded		The panel judged that these were not consistently recorded around the prediction window [22,29] and added limited information beyond laboratory and ABGA markers in our dataset
Neurologic status	GCS, altered mental status [1,7,8]	Excluded		Neurologic status, although reflected in qSOFA, was incompletely documented in the EMRs and overlapped with the criteria used for cohort definition
Clinical chemistry	Total bilirubin, AST, creatinine [1,3]	Retained	Total bilirubin, AST, creatinine	
	Lactic acid [1,3,4,41]		Lactic acid	
	Procalcitonin, sodium, potassium, CRP [3,10,41,46,47]		Procalcitonin, sodium, potassium, CRP	
	BUN, urine output [1,3]	Excluded		Considered redundant with creatinine and acid–base markers and not reliably available at a uniform time point
Hemodynamic / treatment	Vasopressor use [1,3]	Excluded		Reflects treatment decisions and shock status rather than pre-prediction risk and is not consistently available at the prediction time
Respiratory support	Mechanical ventilation [3,4]	Excluded		Represents respiratory failure and clinician decisions; treated as context or outcome rather than a predictor
Hematologic (CBC)	Hb, Hct, RBC, MCV, MCH, MCHC, RDW, WBC, neutrophils, lymphocytes, monocytes, eosinophils, basophils, nRBC, MPV, PDW, NLR, PLR, platelet count [1,3,40,43–46]	Retained	Hb, Hct, RBC, MCV, MCH, MCHC, RDW, WBC, neutrophils, lymphocytes, monocytes, eosinophils, basophils, nRBC, MPV, PDW, NLR, PLR, platelet count	
Acid–base / ABGA	pH, PaO_2_, PaCO_2_, HCO₃⁻, BE-B, BE-ECF, tHb, SBC, TCO_2_ [1,3,48]	Retained	pH, PaO_2_, PaCO_2_, HCO₃⁻, BE-B, BE-ECF, tHb, SBC, TCO_2_	
	PaO_2_/FiO_2_ ratio [1,3,8,26,40–42]		PaO_2_/FiO_2_ ratio	
	PAO_2_, AaDO_2_, a/A ratio, CaO_2_ [1,3,26,40–42,49–51]		PAO_2_, AaDO_2_, a/A ratio, CaO_2_	

CCI score = Charlson comorbidity index; SBP = Systolic blood pressure; MAP = Mean arterial pressure; ABGA = Arterial blood gas analysis; GCS = Glasgow coma scale; qSOFA = quick Sequential organ failure assessment; EMRs = Electronic medical records; AST = Aspartate aminotransferase; CRP = C-reactive protein; BUN = Blood urea nitrogen; CBC = Complete blood count; Hb = Hemoglobin; Hct = Hematocrit; RBC = Red blood cell count; MCV = Mean corpuscular volume; MCH = Mean corpuscular hemoglobin; MCHC = Mean corpuscular hemoglobin concentration; RDW = Red cell distribution width; WBC = White blood cell count; nRBC = Nucleated red blood cell; MPV = Mean platelet volume; PDW = Platelet distribution width; NLR = Neutrophil-to-lymphocyte ratio; PLR = Platelet-to-lymphocyte ratio; pH = Hydrogen ion concentration; PaO_2_ = Arterial oxygen partial pressure; PaCO_2_ = Arterial carbon dioxide partial pressure; HCO₃⁻ = Bicarbonate; BE-B = Base excess in blood; BE-ECF = Base excess in extracellular fluid; tHb = Total hemoglobin; SBC = Standard bicarbonate concentration; TCO_2_ = Total carbon dioxide; PaO_2_/FiO_2_ ratio = Ratio of arterial oxygen partial pressure to fraction of inspired oxygen; PAO_2_ = Calculated alveolar oxygen partial pressure; AaDO_2_ = Alveolar–arterial oxygen difference; a/A ratio = Arterial/alveolar oxygen ratio; CaO_2_ = Arterial oxygen content.

F1-score = Harmonic mean of precision and recall; AUC = Area under the curve.

† Justification reflects the expert panel’s assessment of clinical relevance, redundancy, and data availability for each predictor, and is provided only where variables were modified, added, or excluded relative to the initial candidate list.
